# AdipoCount: A New Software for Automatic Adipocyte Counting

**DOI:** 10.3389/fphys.2018.00085

**Published:** 2018-02-20

**Authors:** Xuhao Zhi, Jiqiu Wang, Peng Lu, Jue Jia, Hong-Bin Shen, Guang Ning

**Affiliations:** ^1^Institute of Image Processing and Pattern Recognition, Shanghai Jiao Tong University, Shanghai, China; ^2^Key Laboratory of System Control and Information Processing, Ministry of Education of China, Shanghai, China; ^3^Department of Endocrinology and Metabolism, China National Research Center for Metabolic Diseases, Ruijin Hospital, Shanghai Jiao Tong University School of Medicine, Shanghai, China; ^4^Laboratory of Endocrinology and Metabolism, Shanghai Institutes for Biological Sciences, University of Chinese Academy of Sciences, Chinese Academy of Sciences, Shanghai, China; ^5^Affiliated Hospital of Jiangsu University, Zhenjiang, China

**Keywords:** cell counting, obesity, adipocytes, image segmentation, software validation, cellularity, AdipoCount

## Abstract

Obesity has spread worldwide and become a common health problem in modern society. One typical feature of obesity is the excessive accumulation of fat in adipocytes, which occurs through the following two physiological phenomena: hyperplasia (increase in quantity) and hypertrophy (increase in size) of adipocytes. In clinical and scientific research, the accurate quantification of the number and diameter of adipocytes is necessary for assessing obesity. In this study, we present a new automatic adipocyte counting system, AdipoCount, which is based on image processing algorithms. Comparing with other existing adipocyte counting tools, AdipoCount is more accurate and supports further manual correction. AdipoCount counts adipose cells by the following three-step process: (1) It detects the image edges, which are used to segment the membrane of adipose cells; (2) It uses a watershed-based algorithm to re-segment the missing dyed membrane; and (3) It applies a domain connectivity analysis to count the cells. The outputs of this system are the labels and the statistical data of all adipose cells in the image. The AdipoCount software is freely available for academic use at: http://www.csbio.sjtu.edu.cn/bioinf/AdipoCount/.

## Introduction

Cell counting is a very common and fundamental task in research and clinical practice. For instance, accurate cell counting is very important in the study of cell proliferation. When pathologists make diagnostic decisions, they want to refer to the number of cells (Kothari et al., 2009).

In the past, one common way to perform this task was to count cells manually with the help of tools such as a counting chamber. However, manual examination and counting is very time-consuming and highly dependent on the skills of operators. With the increasing demand for cellular analysis, labor-intensive manual analysis was gradually replaced by automatic cell counting methods (Landini, 2006; Han et al., 2012). Among the automatic methods, one of the most powerful and versatile methods for cellular analysis is computer image analysis using image processing algorithms.

Many algorithms have been developed for cell segmentation and counting. There exist diversity and specificity among cell morphologies, microscopes and stains; however, most algorithms are specifically designed for one or several types of cells (Di Rubeto et al., 2000; Liao and Deng, 2002; Refai et al., 2003). It is necessary but difficult to develop a generally applicable cell segmentation method (Meijering, 2012). Common cell segmentation approaches are mainly divided into intensity thresholding (Wu et al., 2006), feature detection (Liao and Deng, 2002; Su et al., 2013), morphological filtering (Dorini et al., 2007), deformable model fitting (Yang et al., 2005; Nath et al., 2006), and neural-network-based segmentation (Ronneberger et al., 2015). The most predominant and widely used approach for cell segmentation is intensity thresholding (Wu et al., 2010). In addition, software programs that target biological images have been developed, such as Fiji (Schindelin et al., 2012), CellProfiler (Lamprecht et al., 2007), and Cell Image Analyzer (Baecker and Travo, 2006).

Software programs have developed for semi-automatic or automatic adipose cell counting (Chen and Farese, 2002; Björnheden et al., 2004; Galarraga et al., 2012; Osman et al., 2013; Parlee et al., 2014). For instance, Adiposoft (Galarraga et al., 2012), which is one of the best adipocyte counting software programs, has been developed as a plug-in for Fiji (Schindelin et al., 2012). The process of Adiposoft is very straightforward: First, the red channel of the input image is processed by thresholding. Second, a watershed algorithm is used to segment the adipose cells. In general, most adipocyte counting systems require images to be of high quality, and noise in the image will result in reduced counting accuracy. However, in the production of adipose slices and microscopy imaging, noise is inevitable, and staining quality is mixed. Therefore, the most common way of detecting the proliferation (change in quantity) and hypertrophy (change in diameter) of adipocytes is still manual counting. In contrast to other existing automatic adipose cell counting tools, AdipoCount uses not only gray information but also gradient information to segment the membrane in an adipocyte image. In addition, AdipoCount has a pre-processing step for eliminating noise and correcting illumination and uses a series of post-processing steps to improve the segmentation result. We also develop a re-segmentation step in AdipoCount for dealing with missing dyed membrane and improving the counting accuracy, which is an innovation in adipose cell counting software. Additionally, the segmentation result can be manually corrected by adding or erasing lines on it.

Most cells are cytoplasm-stained or nucleus-stained and the cells are blob-like, which makes it easy to perform intensity thresholding and ellipse fitting. Unlike most cell images, adipose cell images are membrane-stained, which results in large differences in the adipose cell segmentation approach, compared to methods for segmenting other types of cells. To detect all the adipose cells, the stained membrane should be segmented first, which can be used to estimate the interior of each adipose cell. After the segmentation of cells, a connected domain analysis algorithm is executed to count the cells.

The staining quality of slices will affect the accuracy of cell counting. For slices with high staining quality, the segmentation algorithm is very reliable and its counting accuracy is high. However, for poorly stained adipose tissue images, there could be noise due to a minced membrane and miss-staining is common, so it is necessary to use a pre-processing step to eliminate noise and a re-segmentation process to complete missing areas of the membrane. To address uneven stain quality, we design the adipose cell counting system with three modules: an illumination correction module, a pre-processing module for eliminating noise, and a re-segmentation module for completing missing dyed areas of the membrane. The outputs of this system are statistical data for all cells and a visualized counting result (labeled adipose cell image). Based on the statistical data and the visualized counting result, further manual correction can be efficiently performed.

## Methods

**Figure 1:**
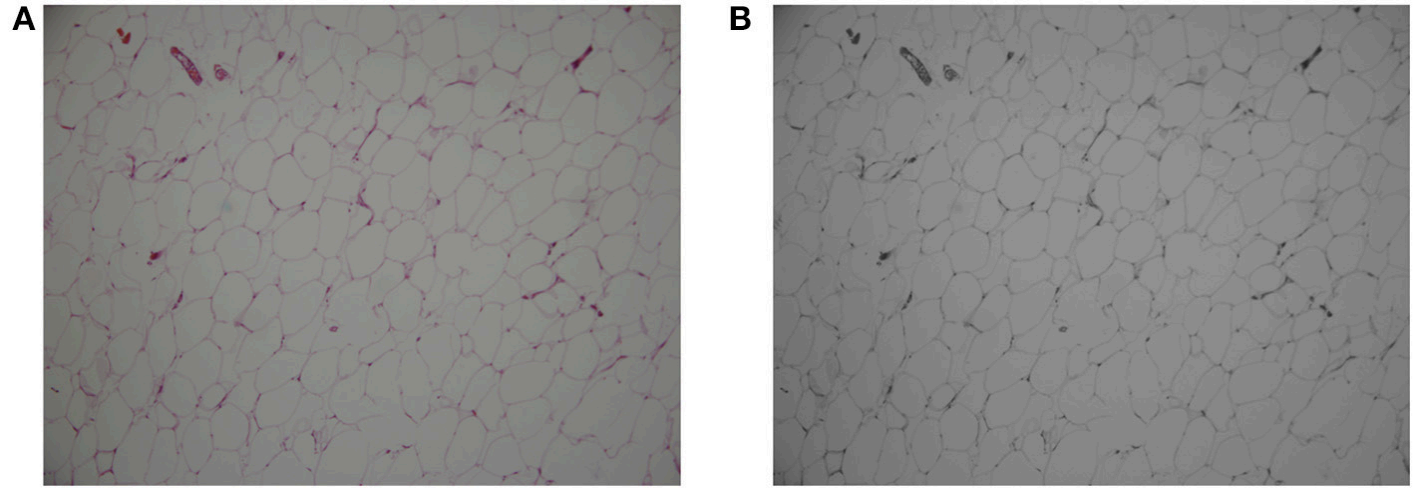
Original color image of adipose cells **(A)** and its grayscale image **(B)**.

For a given stained adipose tissue image, as illustrated in Figure 1, the first step is graying, which outputs a grayscale image. The graying process ignores the color information and retains the intensity information, which is used for further thresholding and edge detection.

**Figure 2:**
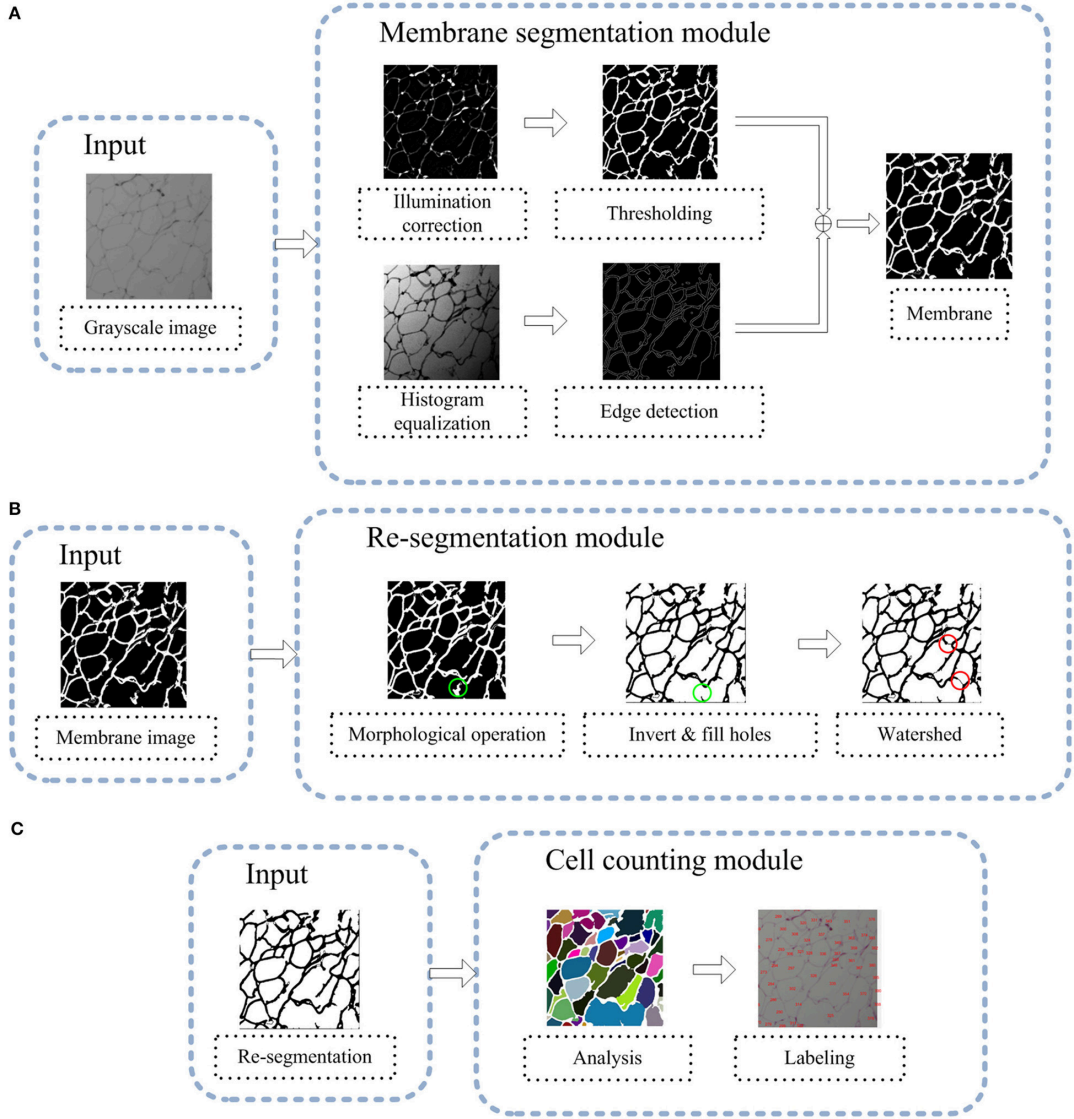
**(A)** Flowchart of the membrane segmentation module. The input is a grayscale image, and the output is a segmented membrane. The binary image that is generated by thresholding and the edge detection result are combined to generate the final membrane segmentation output. **(B)** Flowchart of the re-segmentation module. The input is a segmented membrane image. The morphological operation can fill holes in the membrane and make the segmentation of the membrane more reliable. Then, we invert the image and fill holes inside the cell area; as illustrated by the green circle, the separated and short segments of the membrane are eliminated. Then, a watershed-based algorithm is used, and as indicated by the red circle, some missing dyed areas of the membrane are detected. **(C)** Flowchart of the cell counting module. The input of this module is a re-segmentation image and the image is labeled using a connected-domain analysis method.

As shown in Figure 2, the AdipoCount system contains the following three modules: (1) a membrane segmentation module, (2) a re-segmentation module, and (3) a cell counting module.

The input of the membrane segmentation module is a grayscale image, and there are two parallel processes, namely, thresholding and edge detection. The outputs of these two processes are combined to generate the final membrane segmentation result. Before thresholding and edge detection, an image enhancement step is performed: for thresholding, illumination correction is carried out on the grayscale image; for edge detection, histogram equalization is performed on the grayscale image.

The input of the re-segmentation module is the membrane segmentation result that was generated by the membrane segmentation module. With the membrane image, first, a post-process is implemented to eliminate noise and estimate the interiors of adipose cells. Second, a watershed-based algorithm is used for further image segmentation, which can complete some missing dyed areas of the membrane to improve the final accuracy of cell counting.

The input of the cell analysis module is the re-segmentation result from the re-segmentation module. To perform cell counting, a connected domain analysis method is applied to detect all adipose cells. The final results of the AdipoCount system are statistical data on all adipose cells and the labeled image, which provides a basis for manual verification.

### Membrane segmentation

**Figure 3:**
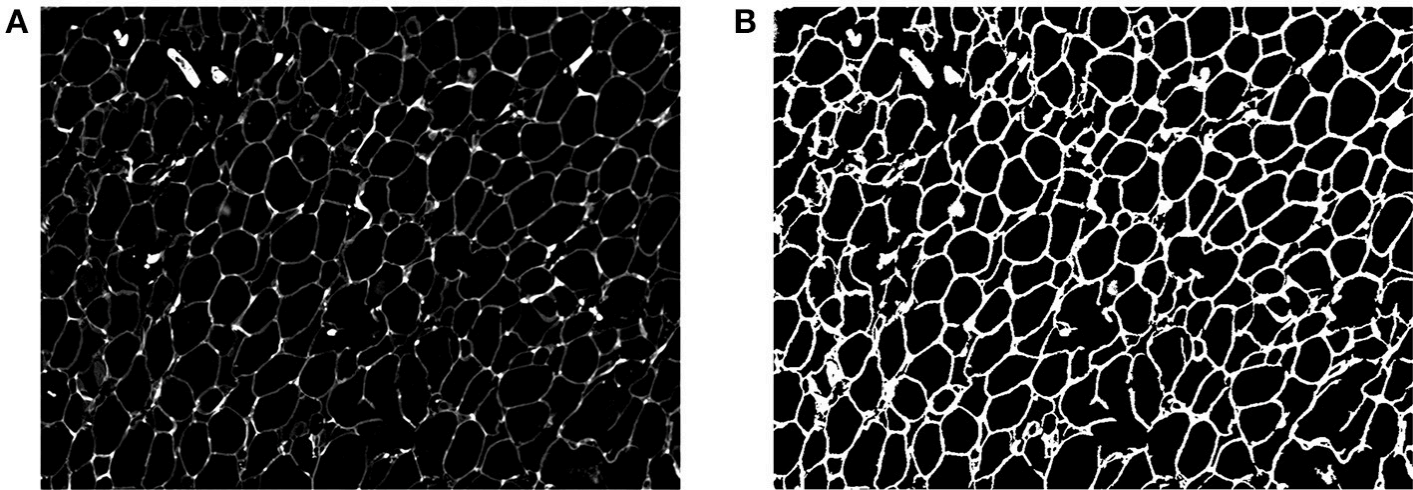
**(A)** The image after illumination correction. **(B)** Binary image after thresholding.

Because the adipose cells are membrane-stained, the entire membrane should be segmented to detect cell interiors. As shown in Figure 2A, in the membrane segmentation module, there are two separate processes: thresholding and edge detection. Thresholding is membrane segmentation according to the pixel intensity, through which the input grayscale image is converted into a binary image. Before thresholding, an illumination correction process is necessary (Leong et al., 2003), because the input image may have uneven illumination since corner areas of an image are always darker than the center area. To implement illumination correction, we define two Gaussian filters, which are denoted as *g*_1_ and *g*_2_, as follows:

(1) $$\begin{array}{r} g_{\sigma }\left(x,\;y\right)=\frac{1}{\sqrt{2\;\pi \;\sigma^{2}}}\text{exp}\left(-\frac{{\left(x-\mu \right)}^{2}+{\left(y-\mu \right)}^{2}}{2\sigma^{2}}\right)\left(-H<x,\;y<H\right) \end{array}$$

where μ is set to zero; σ is the standard deviation of the distribution, which is set to 0.5 for *g*_1_ and 30 for *g*_2_; *H* is the kernel size, which is set to 3 for *g*_1_ and 60 for *g*_2_; and (*x, y*) is the position relative to the center of the window. Then, illumination correction is implemented as follows:

(2) $$\begin{array}{r} I_{c}=I^{*}\left(g_{2}-g_{1}\right) \end{array}$$

where ^*^ stands for convolution, *I* is the input grayscale image and *I*_*c*_ is the illumination-corrected image, as illustrated in Figure 3A. *g*_1_ is a Gaussian filter with a small kernel size, and an image that is blurred by *g*_1_ has some noise filtered out and retains most of the information from the original image. *g*_2_ has a much bigger kernel size than *g*_1_. When using *g*_2_ for convolution, the pixels inside its kernel are blurred, which can make the illumination more equalized. An image that is blurred by *g*_2_ only retains low-frequency information. By the subtraction operation, we can filter the low-frequency information and keep the high-frequency information, such as the membrane.

After the above process, the illumination is much more even. Then, an adaptive thresholding method, namely, OTSU, is used. OTSU (Otsu, 1979) is a classic thresholding method, which can find a threshold that separates foreground from background with maximum intensity variance.

**Figure 4:**
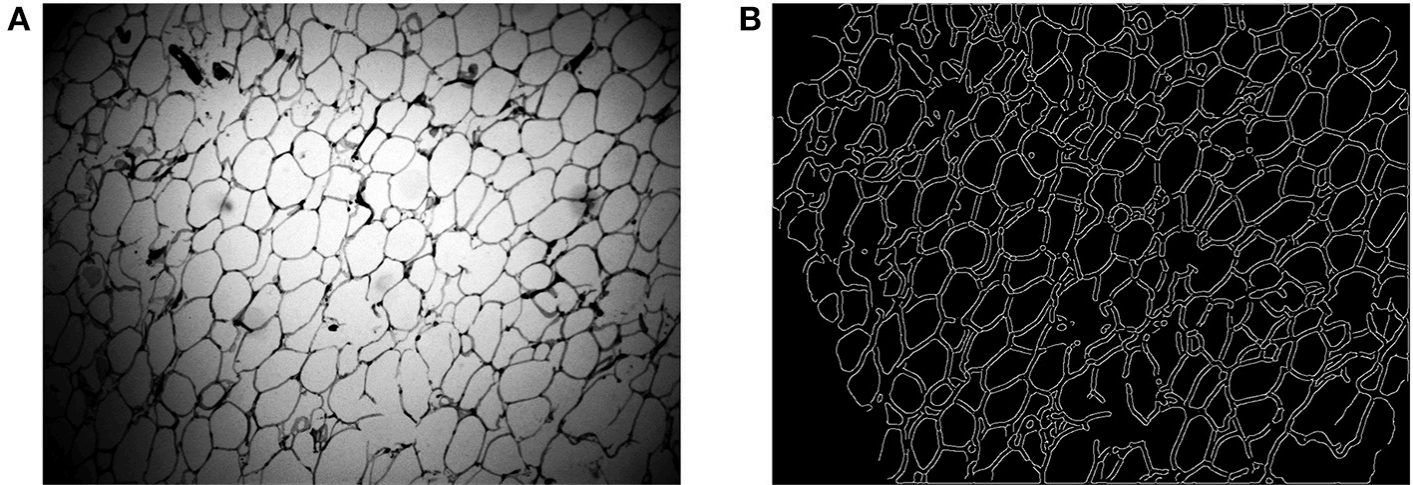
**(A)** The histogram equalization image. **(B)** Membrane edge.

Some areas of the membrane are stained too weakly and the intensity is not strong enough for segmentation by thresholding. Thus, edge detection is also used to ensure that the entire stained membrane can be segmented. Before edge detection, as shown in Figure 4, a histogram equalization process is implemented to enhance the contrast of the image, so the edge can be easily detected. Then, we use the Canny edge detection algorithm (Canny, 1986) to detect the membrane. After thresholding and edge detection, we can obtain the binary image (Figure 3B) and the edge image (Figure 4B). Then, we add those two images together to generate the membrane.

### Re-segmentation

During the processes of slicing and dyeing adipose tissue, minced membrane and other tissues may occur as noise. To address this problem, we develop a post-process for eliminating noise.

**Figure 5:**
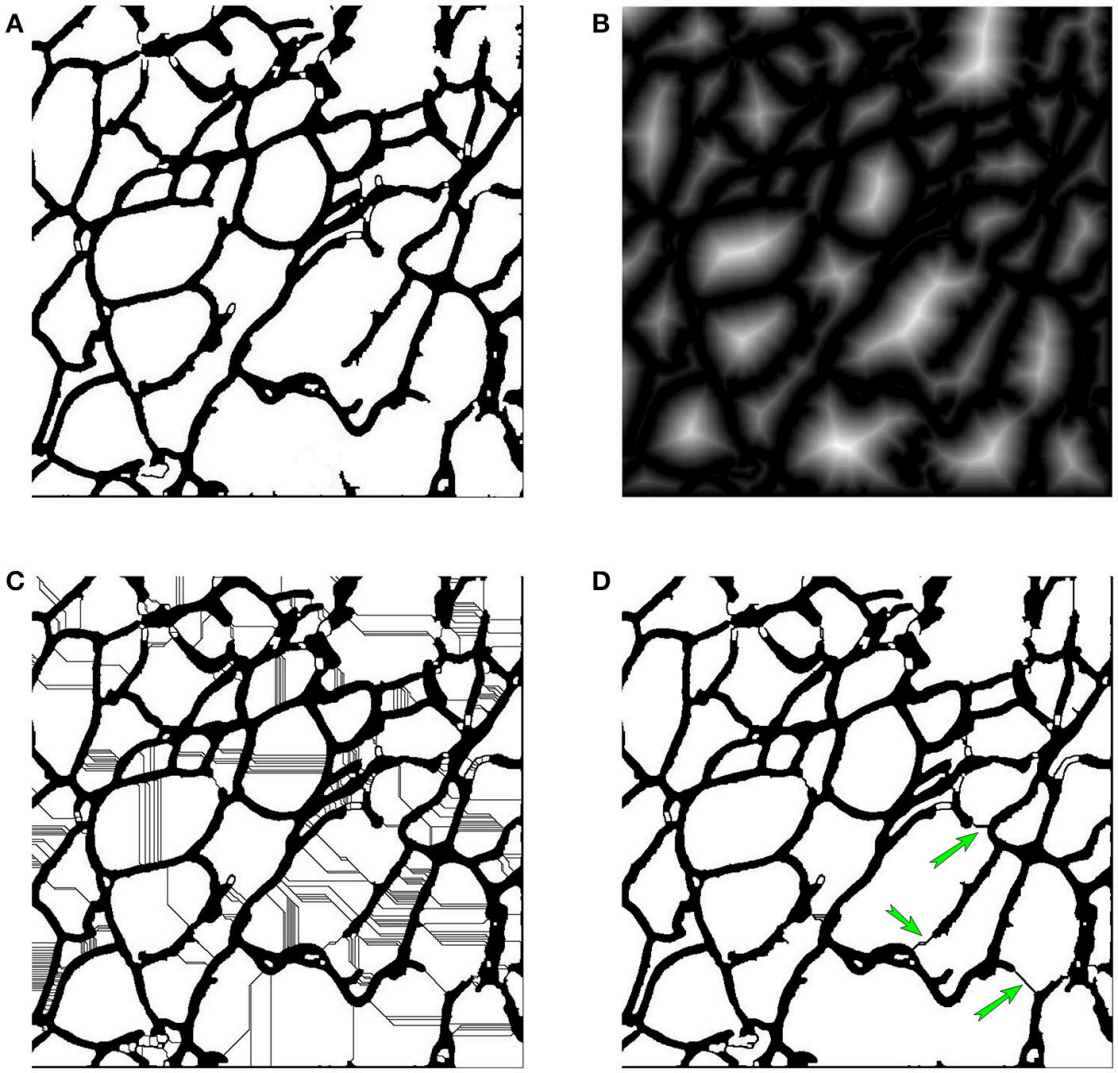
**(A)** The post-processed image. **(B)** Distance map. **(C)** Watershed-transformed image. **(D)** Re-segmented image, where green arrows show the detected missing dyed membrane.

As shown in Figure 2B, the input of this module is a binary image of the membrane. As is well known, in the adipose tissue, adipose cells are closely packed. Thus, in a binary image of the membrane, all membrane segments should be combined into a single connected domain. The noise is always isolated in a small domain, which can be removed by a morphological operation. Specifically, we first detect the entire connected domain and eliminate all subdomains with areas that are smaller than*T*, where *T* is a defined area threshold. Then, an opening operation, a closing operation and a dilation operation are applied sequentially. Next, an intensity reversal operation is applied to invert the background and foreground. Then, we fill the holes inside the cell area, to remove the separated and short membrane areas. The post-processed image is shown in Figure 5A.

After the post-process, some noise can be eliminated, but there remain missing dyed membrane areas, which will lead to inaccurate detection of adipose cells. To estimate the membrane, a watershed-based algorithm is applied for further image segmentation. The watershed algorithm was proposed by Beucher (Beucher and Lantuéjoul, 1979), improved by Vincent and Soille (1991), and has become a popular image segmentation method.

The watershed algorithm stimulates a flooding process. A grayscale image is identified with a topographical surface, in which every point's altitude equals the intensity of the corresponding pixel. Before the watershed transform, we first compute the distance map (Meyer and Fernand, 1994), in which every pixel's value is the distance from the nearest zero-point. The distance map is shown in Figure 5B. Then, we use watershed algorithm to perform further image segmentation on the distance map. Through the watershed transform, many watersheds are generated, as illustrated in Figure 5C, which is over-segmented.

To detect the membrane, we develop a judgement criterion for filtering false-positive watersheds and choose the most-probable watersheds as membrane areas. In our protocol, we consider watershed *w* as a membrane of domain *D*, and split *D* into two sub-domains, which are denoted as *D*_1_ and *D*_2_, if it satisfies both of the following conditions:

(3) $$\begin{array}{r} L<L_{T} \end{array}$$

(4) $$\begin{array}{r} \frac{A_{1}}{A_{2}}<R_{a} \end{array}$$

where *L* is the length of *w* and *L*_*T*_ is the threshold, which can filter long watersheds since missing dyed membrane segments are more likely to be short. *A*_1_ and *A*_2_ are the areas of *D*_1_ and *D*_2_ (*A*_1_ > *A*_2_), and *R*_*a*_ is the area ratio threshold, which ensures that the sub-domains have similar area. After this filtering process, we can obtained the most-probable missing dyed membrane segments, as illustrated in Figure 5D, which are shown with green arrows.

### Cell counting

**Figure 6:**
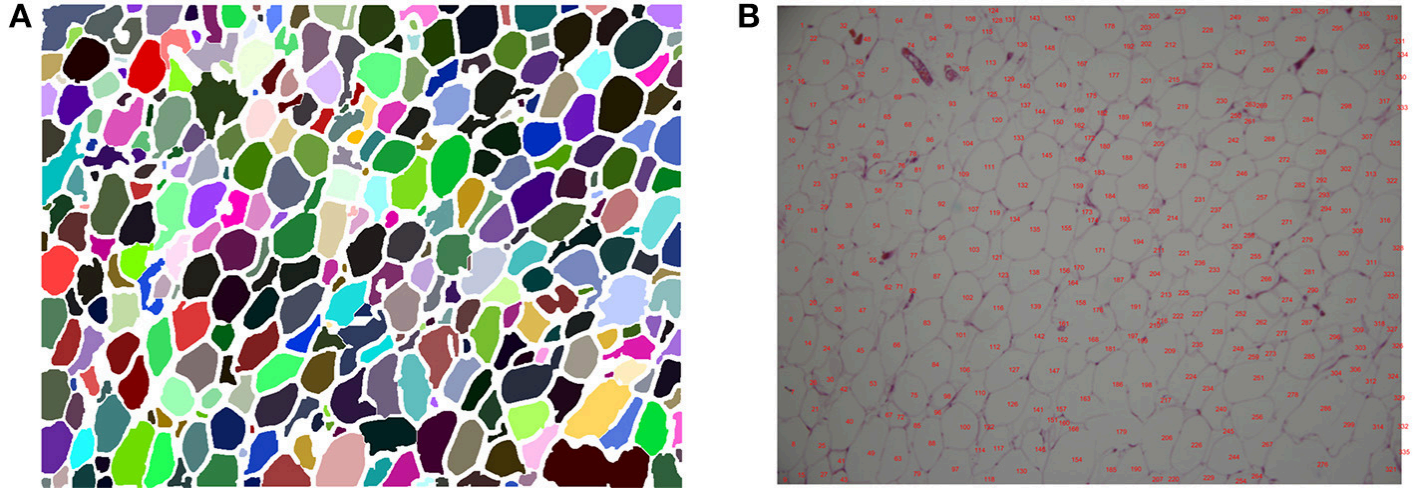
**(A)** Visualized segmentation result, in which each adipose cell is labeled with a different color. **(B)** Labeled image with cell numbers.

After re-segmentation, adipose cells are segmented, so cell counting and analysis can be implemented; the flowchart is shown in Figure 2C. Each adipose cell is a connected domain in the image, so we first detect all the connected domains using the region-growing method (Adams and Bischof, 1994). After this process, we can obtain every cell's position, area and diameter. Then, we label every cell's number at the corresponding position on the original color image; the visualized results are shown in Figure 6. With the statistical data and the labeled image, further correction can be easily performed manually.

We use F1-score to evaluate the counting result. The F1-score is calculated by:

(5) $$\begin{array}{r} P=\frac{TP}{GT} \end{array}$$

(6) $$\begin{array}{r} R=\frac{TP}{EN} \end{array}$$

(7) $$\begin{array}{r} F=2^{*}\frac{P^{*}R}{P+R} \end{array}$$

where *P* is precision, *R* is recall rate, and *F* is F1-score.

## Experiments and discussion

To test the robustness of AdipoCount, we selected a batch of stained adipose tissue images. These images have different dyeing qualities. Images with high quality have clear membranes; those with low quality may have large amounts of noise and many blurred membrane areas. The results show that our system achieves high counting accuracy on high-dyeing-quality images. For low-dyeing-quality images, AdipoCount eliminates most noise and recovers some missing dyed membrane areas, which improves the overall segmentation results.

**Figure 7:**
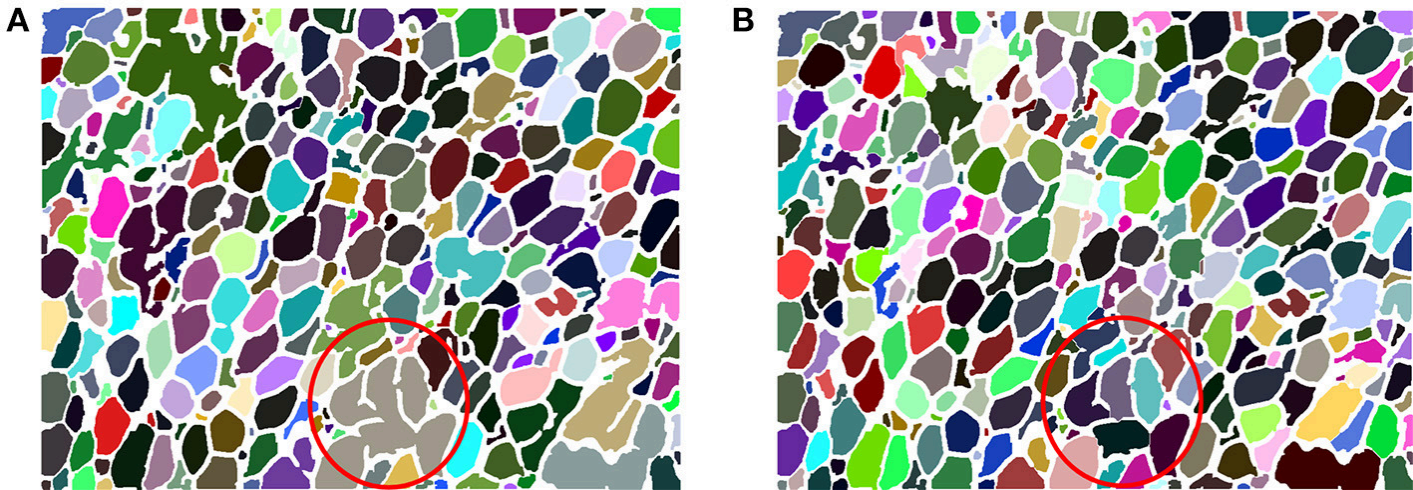
**(A)** AdipoCount's segmentation result without re-segmentation. The number of detected cells is 303. **(B)** AdipoCount's segmentation result with re-segmentation. The number of detected cells is 335.

Due to the constraints of the dyeing process, missing dyed membrane areas are inevitable, so the re-segmentation process is necessary. Figure 7A is the segmentation result without a re-segmentation process and Figure 7B is the segmentation result with a re-segmentation process. Some missing dyed membrane areas are detected by the re-segmentation module (e.g., in Figure 7A, the brown region inside the red circle is segmented into 6 different cells, as shown in Figure 7B). Therefore, more adipose cells can be segmented, which can improve the counting results.

**Figure 8:**
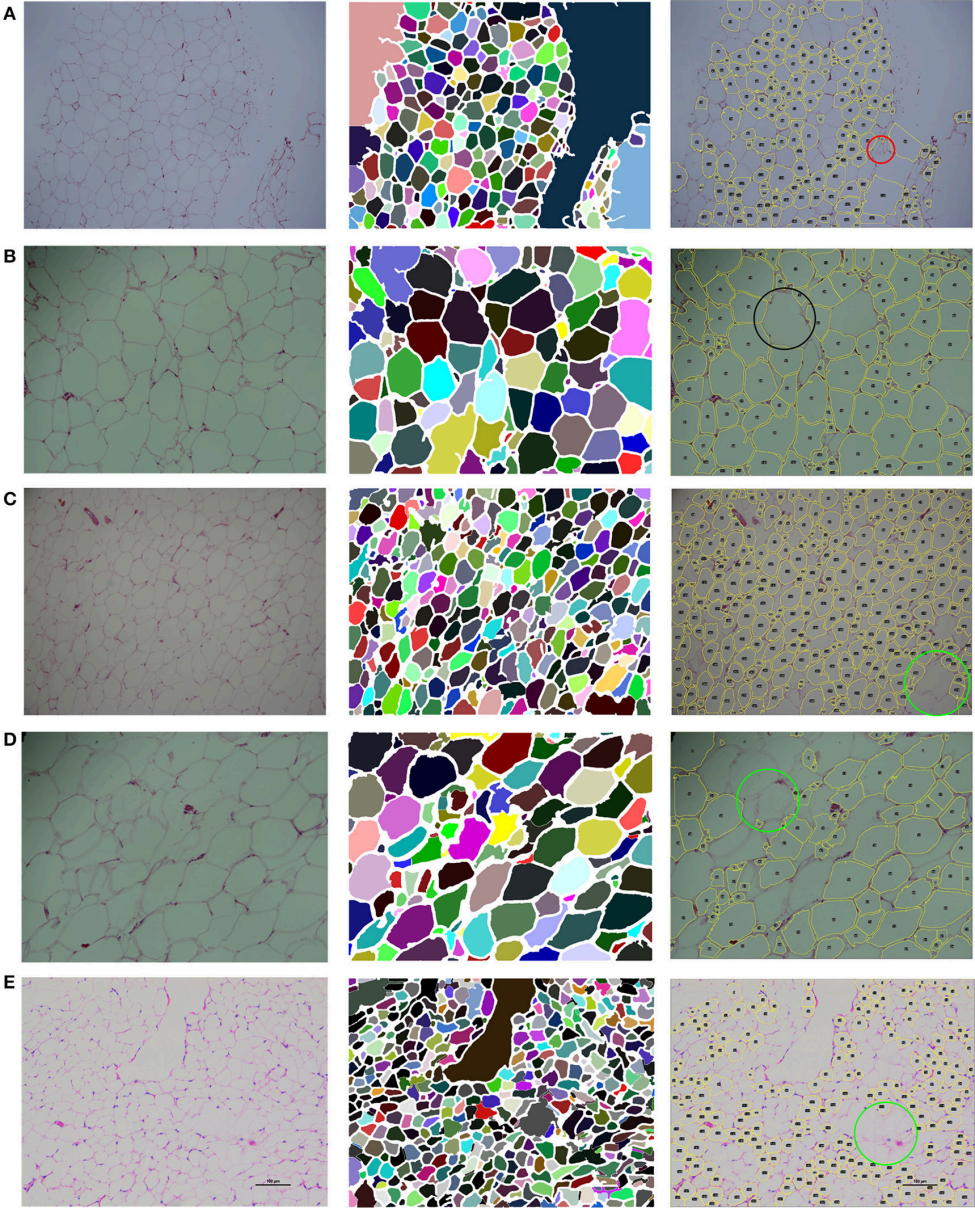
Segmentation of five test images. The segmentation results in the second column are generated by AdipoCount. The segmentation results in the third column are generated by Adiposoft. **(A–C)** Three high-quality stained images and their segmentation results. **(D)** An image with a blurred membrane and its segmentation results. **(E)** An image with dense adipose cells and its segmentation results.

As shown in Figures 8A–C, our segmentation results for high-quality stained images are satisfactory. Nearly the entire membrane is segmented and most adipose cells are correctly detected. For an image with a blurred membrane, as shown in Figure 8D, our system still detects the blurred membrane, which is thicker than a clear membrane. For images with dense adipose cells, such as that shown in Figure 8E, which are usually small, our system detects them with high accuracy.

We further compared our AdipoCount with Adiposoft, which can be download at http://imagej.net/Adiposoft. We used 5 adipocyte images to evaluate the counting accuracies of these two software programs, and the counting results are listed in Table 1. Based on these results, AdipoCount outperforms Adiposoft. As shown in Figure 8, cells are commonly missed during counting in segmentation results that are generated by Adiposoft, some cells with small size (e.g., the region inside the red circle in Figure 8A) or large size (e.g., the region inside the black circle in Figure 8B) are not detected, and cells with blurred membrane are not detected (e.g., the region inside the green circle in Figures 8C–E). We also compare the computation times of these two software programs. As shown in Table 2, our AdipoCount is more efficient than Adiposoft.

**Table 1:** Cell counting results of 5 test images^a^.

**Test image**	**Method**	**GT**	**EN**	**FP**	**FN**	**TP**	**F1-score (%)**
Figure 8A	AdipoCount	266	255	5	16	250	96.0
	Adiposoft		169	12	109	157	72.2
Figure 8B	AdipoCount	128	126	6	8	120	94.5
	Adiposoft		116	12	24	104	85.3
Figure 8C	AdipoCount	354	335	6	25	329	95.5
	Adiposoft		302	45	97	257	78.4
Figure 8D	AdipoCount	120	113	5	12	108	92.7
	Adiposoft		90	14	44	76	72.4
Figure 8E	AdipoCount	475	447	25	53	422	91.5
	Adiposoft		209	27	293	182	53.2

a *GT, ground-truth manual-counting cell number; EN, estimated number of cells that are segmented by the method; FP, false-positive detection; FN, false-negative detection; TP, true-positive detection*.

**Table 2:** Computation times of 5 test images^a^.

**Test Image**	**Figure 8A**	**Figure 8B**	**Figure 8C**	**Figure 8D**	**Figure 8E**
AdipoCount	4.56 s	2.96 s	8.15 s	5.53 s	6.75 s
Adiposoft	12.26 s	10.56 s	11.13 s	11.83 s	7.15 s

a *Hardware: all the experiments were carried out on a PC Intel (R) Core (TM) i7-4790 processor with a clock speed of 3.60 GHz and 8 GB of RAM*.

**Figure 9:**
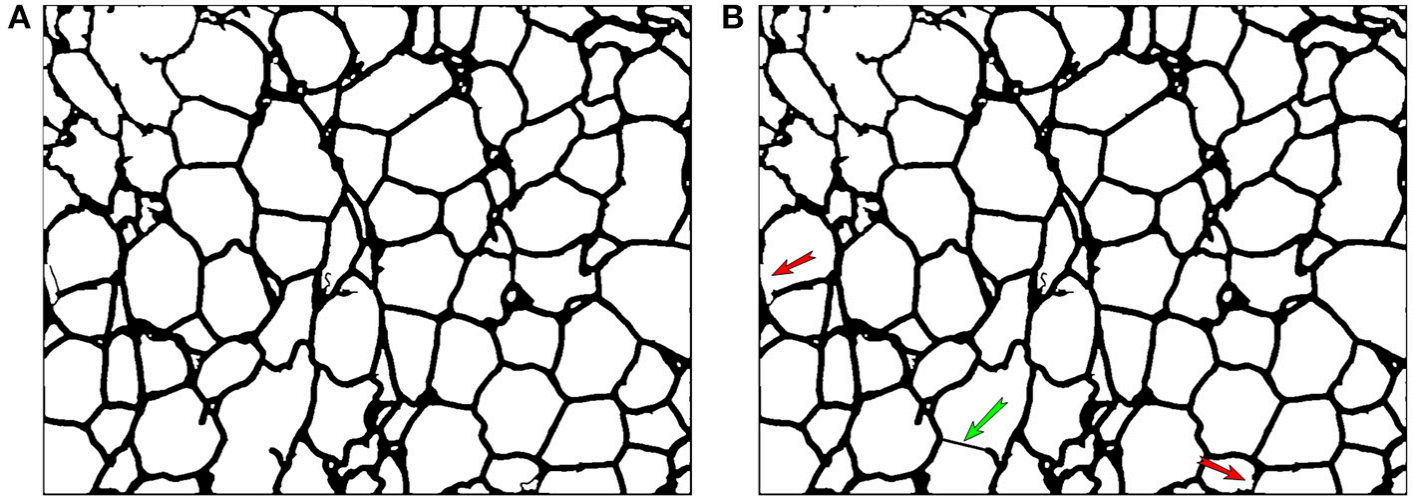
Manual correction of segmentation result. **(A)** The segmentation result before manual correction. **(B)** The manual correction result, where the green arrow shows an added membrane segment, and the red arrows show deleted membrane segments.

As shown in Figure 9, AdipoCount supports manual correction, such as adding or deleting membrane segments in the segmentation result. In addition, manual correction is convenient to implement. We can select two points in the segmentation result (Figure 9A) and draw a line between them to create a new membrane. If we want to delete a membrane, we can draw a rectangle on top of the segmentation result and eliminate the membrane inside it. By manual correction, AdipoCount can add missing membrane segments and delete false membrane segments, which results in improved counting accuracy.

## Conclusions

Adipose cell counting is an important task in obesity research. Multiple software programs have been developed for biological image processing, such as Fiji, which can aid biologists in manual adipose cell counting. In addition, researchers have developed automatic adipose cell counting tools, such as Adiposoft, which can process images and generate counting results. However, the counting accuracy and usability of these tools need to be improved. We have developed a fully automatic adipose cell counting system, namely, AdipoCount, which contains three modules: a membrane segmentation module, a re-segmentation module and a cell counting module. The membrane segmentation module can segment the membrane, the re-segmentation module can estimate missing dyed membrane areas and improve the segmentation result, and the cell counting module can detect, count and label the cells in the image. Comparing with other automatic adipose cell counting software programs, AdipoCount uses more image attributes, such as color intensity and gradient information, to generate accurate segmentation results and a watershed re-segmentation method is implemented to address missing dyed membrane areas. The segmentation results can be further corrected manually. AdipoCount has been tested on a batch of adipose tissue images with different dyeing qualities and our results show that AdipoCount outperforms existing software and can provide reliable counting results for reference in clinical studies. However, when applying our system on images with low signal-to-noise ratio (SNR) or many missing dyed membrane areas, the counting results have much room for improvement. In future work, we will add more noise elimination engines, try to improve AdipoCount's robustness and efficiency, and enhance its interactive functions to make it more powerful on challenging images.

## Author contributions

XZ, JW, PL, JJ, H-BS, and GN discussed the project idea. XZ and H-BS designed and developed the method. XZ, JW, PL, JJ, and GN discussed and verified the tested data. Under H-BS and GN instructions, XZ did the experiments, and the paper was written by XZ, JW, and H-BS.

### Conflict of interest statement

The authors declare that the research was conducted in the absence of any commercial or financial relationships that could be construed as a potential conflict of interest.
